# Roux‐en‐Y gastric bypass surgery decreases intestinal aryl hydrocarbon receptor signaling in male mice

**DOI:** 10.14814/phy2.70524

**Published:** 2025-08-26

**Authors:** Annie Wang, Madelynn E. Agabao‐Tucker, Michael L. Goodson, Allison K. Ehrlich, Bethany P. Cummings

**Affiliations:** ^1^ Department of Surgery, Center for Alimentary and Metabolic Sciences, School of Medicine University of California – Davis Sacramento California USA; ^2^ Department of Molecular Biosciences, School of Veterinary Medicine University of California – Davis Davis California USA; ^3^ Department of Anatomy, Physiology, and Cell Biology, School of Veterinary Medicine University of California – Davis Davis California USA; ^4^ Department of Environmental Toxicology University of California – Davis Davis California USA

**Keywords:** AhR signaling, bariatric surgery, tryptophan metabolites

## Abstract

The impact of Roux‐en‐Y gastric bypass (RYGB) on indole metabolism and AhR signaling is poorly understood. Therefore, we tested the hypothesis that RYGB changes the indole metabolite profile in the gut, leading to changes in AhR signaling. To test this hypothesis, we developed a mouse model of RYGB that recapitulates the human procedure. Given that indole metabolism is influenced by body weight, we included sham‐operated control mice that were either fed ad libitum (S‐AL) or food restricted to match body weight to RYGB (S‐WM). We measured weight‐ and surgery‐dependent changes to glucose metabolism, indole metabolism, and AhR activation. RYGB‐operated mice exhibited body weight‐independent improvements in glucose tolerance, insulin, and glucagon‐like peptide‐1 secretion. Plasma levels of indole‐3‐propionic acid (I3PA) and kynurenic acid (KA) were reduced in RYGB mice compared to S‐WM mice, but not S‐AL mice. However, the S‐WM group exhibited a trend for increased I3PA and KA compared to S‐AL. Consistent with the reduction in indoles, RYGB reduced AhR signaling and increased lipocalin‐2 expression, a marker of inflammation. These data suggest that indole metabolites do not contribute to the metabolic benefits of RYGB, but their reduction may contribute to increases in gut inflammation after RYGB.

## 1 INTRODUCTION

Bariatric surgery, including Roux‐en‐Y gastric bypass (RYGB), remains the most effective and durable treatment option for obesity and produces robust metabolic improvements (Courcoulas et al., 2018). RYGB induces changes in nutrient intake and transit and alters multiple facets of gut physiology as well as gut microbiota composition (Sandoval & Patti, 2023). Postoperative changes in the gut microbiome have been implicated in the metabolic benefits of bariatric surgery. However, the mechanisms involved remain incompletely defined. In particular, the gut microbiome influences host health, in large part, through the metabolites it produces. Indole metabolites produced by the gut microbiome are implicated in intestinal homeostasis (Borghi et al., 2020; Grifka‐Walk et al., 2021); however, the impact of bariatric surgery on indole metabolism has not been well studied. Herein, we apply a novel mouse model of RYGB to define the impact of RYGB on indole metabolism.

The gut microbiome influences host health, in part, through the production of metabolites that enter the host systemic circulation to regulate downstream metabolic and inflammatory pathways. Gut microbiome composition is impacted by both obesity (Aron‐Wisnewsky et al., 2021) and surgically‐induced weight loss (Sandoval & Patti, 2023; Yadav et al., 2023). For example, after RYGB, *Bacteroidetes*, *Prevotella*, *Lactobacillus*, *Bifidobacterium*, and *Enterococcus* species have been reported to increase (Geng et al., 2022). Changes to the gut microbiome may contribute to the metabolic benefits of bariatric surgery. Specifically, gut microbiome transfer from rodent models of bariatric surgery (Liou et al., 2013) and from human subjects that underwent bariatric surgery (Anhê et al., 2023; Tremaroli et al., 2015) into germ‐free recipients recapitulates the metabolic benefits of bariatric surgery. However, the mechanisms by which the gut microbiome contributes to changes in host health after bariatric surgery are not well‐defined.

Indoles are a diverse and abundant class of metabolites derived from tryptophan that impact host metabolic health and inflammation. Tryptophan metabolism occurs via: (1) the kynurenine pathway, which produces kynurenic acid (KA) or quinolinic acid; (2) the serotonin pathway, in which serotonin and melatonin are formed; or (3) the indole pathway, in which indole and indole derivatives are formed (Geng et al., 2022; Roth et al., 2021). The initial rate‐limiting step of the kynurenine pathway is catalyzed by indoleamine 2,3‐dioxygenase in the brain, gastrointestinal tract, and the liver (Roth et al., 2021). The indole pathway is mediated by the gut microbiome, which expresses enzymes that catalyze this process (Roth et al., 2021). Specifically, indoles are produced by *E. coli*, *Lactobacillus*, and *Bacteroides* species (Geng et al., 2022). Several tryptophan metabolites, including indole‐3‐lactic acid, indole‐3‐aldehyde, indole‐3‐acetic acid, and indole‐3‐propionic acid, activate the aryl hydrocarbon receptor (AhR) with resultant anti‐inflammatory effects (Geng et al., 2022; Lavelle & Sokol, 2020; Liu et al., 2021).

AhR is expressed in intestinal epithelial cells, intestinal immune cells, and the enteric nervous system (Stockinger et al., 2021). When bound by ligands, including the aforementioned tryptophan metabolites, AhR dimerizes with the AhR nuclear translocator (ARNT) to then bind and activate the AhR response element (AHRE) in the promoter regions of AhR‐responsive genes, such as CYP1A1 (Dong & Perdew, 2020). In intestinal epithelial cells, AhR activation results in regulation of crypt stem cells and epithelial cell turnover, as well as stabilization of tight junctions and maintenance of barrier integrity (Choi et al., 2017; Metidji et al., 2018). AhR activation in intestinal immune cells increases regulatory T cells (Foxp3^+^ and Tr1 cells), downregulation of Th1 and Th17 cells, and increased intestinal homing of regulatory T cells (Sandoval et al., 2023; Stockinger et al., 2021; Xiong et al., 2020). AhR also plays a role in enteric nervous system maturation and intestinal motility (Chen et al., 2023; Obata et al., 2020; Stockinger et al., 2021). However, the impact of bariatric surgery on indole metabolism and AhR signaling is poorly understood. Therefore, we tested the hypothesis that RYGB changes indole metabolite profile in the gut, leading to changes in AhR signaling.

To test this hypothesis, we developed a mouse model of RYGB that recapitulates the human procedure as well as the metabolic benefits of the surgery. Critically, this mouse model allowed us to differentiate weight loss‐dependent and weight loss‐independent changes to indole metabolites, which are difficult to control in human surgery patients. This control is necessary given that obesity influences gut microbial indole metabolism (Agus et al., 2021; Turpin et al., 2023). Herein, we find that key immunoregulatory indoles are decreased after RYGB, in a weight loss‐independent manner. This was associated with a drastic reduction in intestinal AhR signaling and an increase in a marker of intestinal inflammation. Together, these data suggest that indole metabolites do not contribute to the metabolic benefits of RYGB, but may contribute to increases in gut inflammation after RYGB.

## 2 METHODS

### 2.1 Animals and procedures

Two‐month‐old male C57BL/6J mice were maintained on a high‐fat diet consisting of ground chow (5012 LabDiets; St. Louis, MO) supplemented with 3.4% butter fat, 8.5% tallow, 13.1% soybean oil, 3.5% mineral mix, and 1% vitamin mix (Dyets; Bethlehem, PA) by weight for 2 months to produce an obese insulin‐resistant phenotype. Study mice were individually housed and maintained in a temperature‐ and humidity‐controlled room, with a 14:10‐h light–dark cycle. At 4 months of age, mice underwent sham or RYGB surgery. RYGB was performed similarly to the procedure described by Kucharczyk et al. (Kucharczyk et al., 2013) with additional modifications to mimic the anatomic alterations present in human RYGB. After laparotomy, the biliopancreatic limb was transected 3 cm distal to the ligament of Trietz. A 3 cm roux limb was measured distal to the biliopancreatic limb, and an end‐to‐side jejunojejunostomy was created between the biliopancreatic and roux limbs. Next, a gastrojejunostomy was created between the proximal roux limb and the anticipated gastric pouch, which was then created by transecting the stomach and closing the distal and proximal ends. Sham surgeries were performed by making a laparotomy incision and making the same incisions as in the RYGB, but gut segments were reattached in their original anatomic position. Sham‐operated mice were either fed ad libitum (S‐AL) or food restricted to match body weight to the RYGB‐operated mice (S‐WM) (*n* = 5–7 per group). Pre‐ and post‐operative care was conducted as previously described (Garibay et al., 2019). Samples were processed for several distinct studies; all mice with available tissue, cecal, and/or plasma samples were included in the current study.

Oral glucose tolerance testing (OGTT) was conducted 1 month after surgery, as previously described, using 1 g/kg body weight dextrose gavage (Garibay et al., 2019). Blood glucose was measured by glucometer. Serum insulin and total GLP‐1 concentrations were measured by multiplex ELISA in a subset of mice (Meso Scale Discovery). Two months after surgery, mice were fasted overnight and euthanized by pentobarbital overdose for tissue collection. All animal procedures were approved by the Cornell University and UC Davis Animal Care and Use Committees.

### 2.2 Quantification of tryptophan metabolites in mouse plasma and cecal contents

Cecal and plasma tryptophan metabolites were quantified by UPLC‐QTOF‐MS based on the previously described method in a subset of mice (Dong et al., 2020). Plasma samples were extracted in methanol containing indole‐3‐acetic acid‐d4 and kynurenic acid‐d5, dried, and dissolved in 10% acetonitrile containing 1 μM chlorpropamide prior to UPLC‐QTOF‐MS analysis (Dong et al., 2020). Importantly, MS data was collected in multiple reaction monitoring (MRM) mode, and the mass accuracy was calibrated by calibration solution purchased from SCIEX (Framingham, MA).

### 2.3 Real‐time PCR

RNA isolation from jejunal samples was performed using the RNeasy mini kit (Qiagen; Cat # 74104). cDNA was synthesized using the High‐Capacity cDNA Reverse Transcription Kit (Applied Biosystems; Cat # 4368814). qPCR reactions were performed using SYBR™ Green PCR Master Mix (Applied Biosystems; Cat # 4309155). *Cyp1a1* (forward GGTGGCTGTTCTGTGAT, reverse AAGTAGGAGGCAGGCACA) and *Lcn2* (forward ATGTCACCTCCATCCTGGTCAG, reverse GCCACTTGCACATTGTAGCTCTG) levels were normalized to *Actb* (forward AATCGTGCGTGACATCAA, reverse GCCATCTCCTGCTCGAAG) using primers from Integrated DNA Technologies.

### 2.4 Statistics

Body weight data were analyzed by two‐factor ANOVA, and adiposity and OGTT AUCs were analyzed by one‐factor ANOVA with Bonferroni post‐hoc test. Metabolite concentrations were analyzed using repeated measures two‐way mixed effect model (surgery × metabolite) followed by Dunnett's multiple comparison post hoc test. Statistical analysis was performed using GraphPad Prism software (version 10.0.0 for Windows, GraphPad Software, Boston, MA). All data are expressed as mean ± SEM. Differences were considered significant at *p ≤* 0.05.

## 3 RESULTS

### 3.1 RYGB improves glucose regulation independently of body weight in mice

**FIGURE 1:**
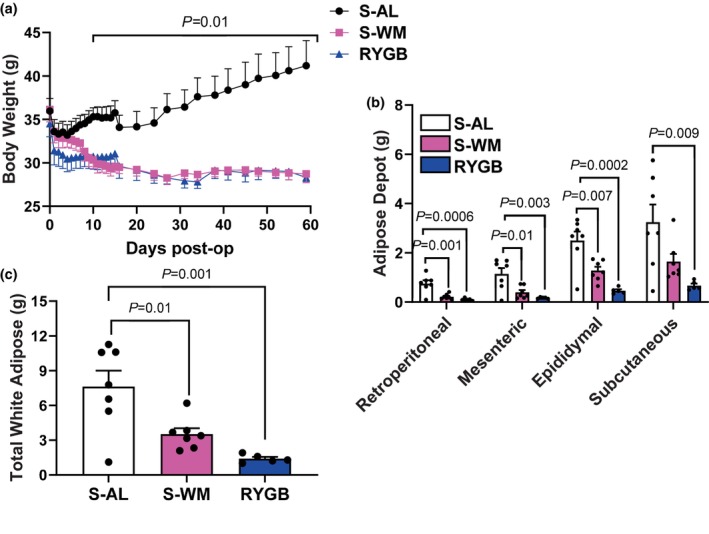
RYGB decreases body weight and adiposity. (a) Body weight, (b) adipose depot weights, and (c) total white adipose weight. Significance determined by two‐factor or one‐factor ANOVA with Bonferroni post‐test, *n* = 5–7.

To determine the impact of RYGB on indole metabolism, we generated a mouse model of RYGB that closely reflects the anatomical alterations and metabolic outcomes observed in clinical practice. As a surgical control, we generated a sham‐operated procedure that recapitulates the surgical manipulations generated in RYGB without altering the gastrointestinal anatomy to control for the effect of surgery. The sham‐operated mice were further divided into two control groups; one group was fed ad libitum (S‐AL) and the other was food restricted to match their body weight to the RYGB group (S‐WM) to control for body weight‐dependent effects. RYGB substantially lowered body weight compared with the S‐AL group (Figure 1a, *p* < 0.05). Further, S‐WM mice were successfully weight matched to RYGB‐operated mice throughout the duration of the study. The reduction in body weight after RYGB was driven by a reduction in adiposity, as adipose depot weight and total white adipose tissue weight were lower in RYGB compared with the S‐AL group (Figure 1b,c, *p* < 0.01). The S‐WM group exhibited similar reductions in visceral adipose depot weights (retroperitoneal, mesenteric, and epididymal) and total white adipose weight compared to the S‐AL group (Figure 1b,c, *p* < 0.05).

**FIGURE 2:**
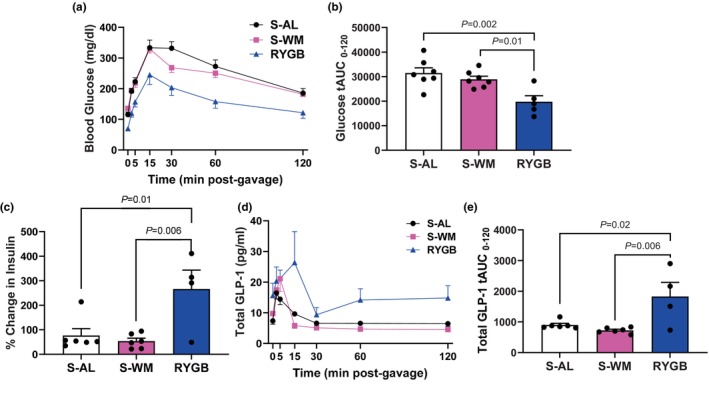
RYGB improves glucose tolerance and increases insulin and GLP‐1 secretion. (a) Blood glucose concentrations and (b) glucose area under the curve (AUC) during an oral glucose tolerance test (OGTT). (c) Percent change in plasma insulin concentration during an OGTT. (d) Serum total GLP‐1 concentrations and (e) AUC during the OGTT. Significance determined by one‐factor ANOVA with Bonferroni post‐test; *n* = 4–7.

To determine the impact of RYGB on glycemic and endocrine regulation, we performed an oral glucose tolerance test (OGTT). RYGB‐operated mice exhibited improvements in glucose tolerance compared with both S‐AL and S‐WM groups (Figure 2a,b, *p* < 0.05). Further, the percent change in circulating insulin in response to the glucose challenge was higher in the RYGB group compared to the S‐AL and S‐WM groups (Figure 2c, *p* < 0.05). Finally, RYGB‐operated mice exhibited increased GLP‐1 secretion in response to the glucose gavage compared with both the S‐AL and S‐WM groups (Figure 2d,e, *p* < 0.05). Together, these data demonstrate that our RYGB mouse model recapitulates the effects of RYGB observed in human patients to improve glucose tolerance, insulin secretion, and GLP‐1 secretion (Holter et al., 2017). Further, these data demonstrate that RYGB improves glucose tolerance, increases insulin secretion, and increases GLP‐1 secretion independently of body weight.

### 3.2 RYGB decreases indole metabolite concentrations independently of body weight in mice

**FIGURE 3:**
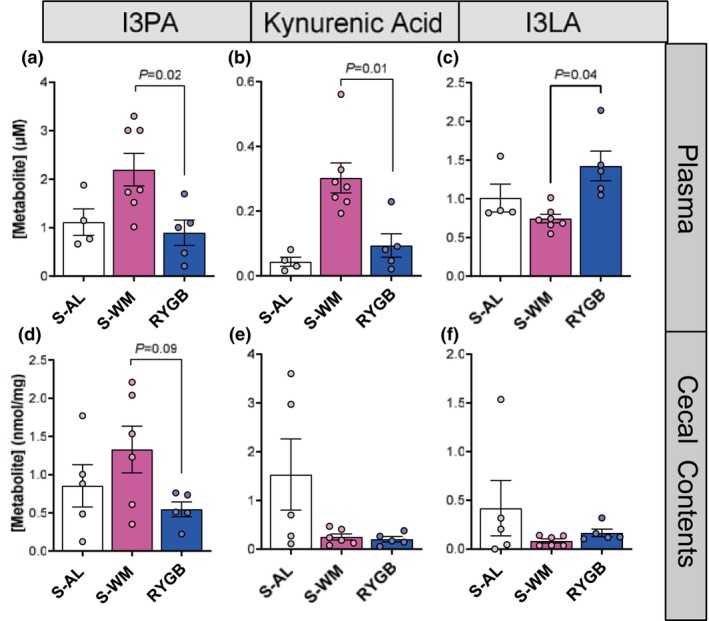
RYGB reduces indole concentrations. Plasma concentrations of (a) indole‐3‐propionic acid (I3PA), (b) kynurenic acid (KA), and (c) indole‐3‐lactic acid (I3LA). Gut luminal concentrations of (d) I3PA, (e) KA, and (f) I3LA. Significance determined by repeated measures two‐factor mixed effect model (surgery × metabolite) with Dunnett's post‐test; *n* = 4–7.

Using our established model of RYGB, we next determined the impact of RYGB on indole metabolism. We analyzed the concentration of indole metabolites in the plasma and gut luminal contents to assess both gut microbial indole metabolism and how this impacts circulating profiles at 2 months after surgery (Figure 3 and Tables S1 and S2). Fasting plasma concentrations of indole‐3‐propionic acid (I3PA) were lower in the RYGB‐operated mice compared to the S‐WM group (Figure 3a, *p* < 0.05). Interestingly, while plasma levels of KA were lower in the RYGB group compared to the S‐WM group (Figure 3b, *p* < 0.05), the plasma concentration of indole‐3‐lactic acid (I3LA) was higher in the RYGB group compared to S‐WM (Figure 3c, *p* < 0.05). Oral administration of I3LA in a DSS‐induced colitis model has previously been shown to activate the AhR pathway and improve intestinal mucosal integrity (Wang et al., 2024). Notably, these changes were in opposition to the effect of food restriction. S‐WM mice tended to have increased plasma I3PA and KA concentrations compared to S‐AL controls. I3PA is solely produced by the gut microbiome, whereas KA is produced by both the host and gut microbiome, which may account for compartment differences. Similar to circulating levels, gut luminal I3PA concentrations tended to be lower in RYGB‐operated mice compared with the S‐WM group (Figure 3d, *p* = 0.09). Gut luminal KA and I3LA concentrations tended to be lower in the RYGB and S‐WM groups, although this did not reach statistical significance (Figure 3e,f, *p* < 0.05). Together, these data demonstrate that RYGB lowers circulating concentrations of I3PA and KA, changes which tend to be reflected in the gut luminal compartment.

### 3.3 RYGB decreases AhR signaling

**FIGURE 4:**
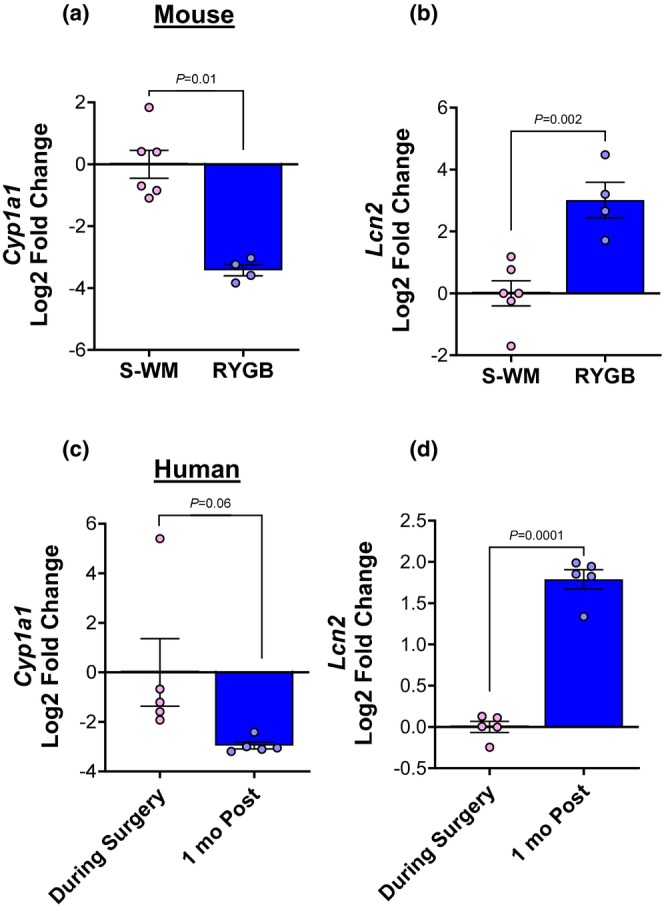
RYGB decreases intestinal AhR signaling. *Cyp1a1* and *Lcn2* mRNA expression in mouse (a, b) and human (c, d) jejunum. Significance determined by *t*‐test; *n* = 4–6.

Given these reductions in indole metabolites, we next assessed whether there were corresponding changes in intestinal AhR signaling. Activation of AhR directly induces expression of *Cyp1a1* by binding to AHRE in the *Cyp1a1* promoter. Therefore, *Cyp1a1* is a commonly used biomarker of AhR signaling. Given that we only observe significant differences in indole concentrations between RYGB and S‐WM, we focused on AhR signaling in these two groups. Consistent with a reduction in known AhR‐regulating indoles, RYGB caused a ten‐fold decrease in intestinal *Cyp1a1* mRNA expression (Figure 4a, *p* < 0.05). *Lcn2* is an innate immune protein that plays a role in iron homeostasis, gut inflammation, and microbial infection (Lu et al., 2019; Moschen et al., 2017). RYGB increased *Lcn2* expression nine‐fold (Figure 4b, *p* < 0.05). The same pattern of decreased *Cyp1a1* and increased *Lcn2* was observed in human jejunum biopsies taken at the time of RYGB and 1 month following surgery (Figure 4c,d; based on publicly available gene expression data GEO Accession # GSE113819; (Ben‐Zvi et al., 2018)). The alteration in intestinal indoles, specifically the reduction in I3PA and KA, following RYGB may explain the drastic reduction in AhR signaling and increased expression of *Lcn2* in the intestine.

## 4 DISCUSSION

Herein, we developed and validated a new mouse model of RYGB which demonstrates reductions in body weight and adiposity along with body weight‐independent improvements in glucose tolerance, insulin secretion, and GLP‐1 secretion. Based on previous work showing that the metabolic syndrome is associated with decreased gut microbial indole production, we expected that RYGB would increase indole metabolites and AhR activation (Natividad et al., 2018). However, we found that RYGB decreased plasma KA and I3PA concentrations in a weight loss‐independent manner. While we found an effect of RYGB to increase plasma I3LA concentrations, RYGB decreased a marker of gut AhR signaling and increased a marker of gut inflammation, supporting an overall decrease in gut AhR signaling after RYGB. These data shed new light on the interplay between bariatric surgery and indole metabolism and suggest that indole metabolites do not contribute to the metabolic benefits of RYGB, but may contribute to increases in gut inflammation after RYGB.

Our findings are consistent with prior studies of kynurenic acid pathway changes after bariatric surgery. A 2022 study of human patients demonstrated that RYGB significantly decreased serum concentrations of KA, tryptophan, quinolinic acid, xanthurenic acid, and 3‐hydroxykynurenine. Additionally, tryptophan, KA, and xanthurenic acid were positively correlated with body mass index and serum hemoglobin A1C, a biomarker of type 2 diabetes mellitus (Yeung et al., 2022). Similarly, among human patients who underwent biliopancreatic diversion/duodenal switch, serum levels of tryptophan, KA, kynurenine, anthranilic acid, 3‐hydroxykynurenine, xanthurenic acid, 3‐hydroxyanthranilic acid, and quinolic acid were significantly decreased up to 12 months postoperatively. Further, prior studies have demonstrated changes in the gut microbiome after RYGB, including enrichment of indole metabolite‐producing *Bacteroides* and *Lactobacillus* species (Tremaroli et al., 2015). While markers of circulating inflammation were not measured in this study, prior work reports that bariatric surgery decreases circulating markers of inflammation. For example, a previous study found that bariatric surgery decreases circulating C‐reactive protein (Christensen et al., 2018).

Obesity is associated with systemic low‐grade inflammation that promotes metabolic disease (Hildebrandt et al., 2023) whereas bariatric surgery decreases systemic inflammation to improve metabolic health (Zhang et al., 2018). However, this relationship changes in the context of gastrointestinal inflammation. Obesity increases intestinal inflammation; however, bariatric surgery has also been reported to increase intestinal inflammation (Leyderman et al., 2023). Moreover, we have previously found that bariatric surgery in mice increases susceptibility to dextran sodium sulfate (DSS)‐induced colitis in mice (Garibay et al., 2019). Notably, human serum kynurenine metabolites are positively associated with some markers of inflammation, such as IL‐10 and neopterin, but negatively associated with other markers, such as TNFα (Deac et al., 2016).

The underlying mechanisms by which bariatric surgery alters gut inflammation are poorly understood. We find that RYGB reduces circulating levels of KA and I3PA, which are associated with a decrease in a marker of intestinal AhR signaling and an increase in a marker of intestinal inflammation. Gut luminal AhR ligands, produced from bacteria, activate AhR to promote gut homeostasis; thus, AhR signaling has a protective role in the gut (de Vos et al., 2022; Stockinger et al., 2021). The decrease in AhR signaling in the RYGB model is in alignment with other models of intestinal inflammation (Hou et al., 2023; Pernomian et al., 2020; Scott et al., 2020). In murine models of inflammatory bowel disease, a deficiency in AhR signaling increased disease progression, inflammatory responses, and epithelial damage (Lamas et al., 2016). Consistent with these studies, it is well established that AhR agonists of microbial, dietary, or xenobiotic origin prevent inflammatory bowel disease (Abron et al., 2018; Benson & Shepherd, 2011; Islam et al., 2017; Kawai et al., 2017; Riemschneider et al., 2021; Scott et al., 2020). Therefore, our data suggest that reductions in AhR signaling may contribute to increases in gastrointestinal inflammation after RYGB.

Overall, there is a complex interplay between obesity, bariatric surgery, and gut inflammation. Herein, we find that RYGB decreases circulating KA and I3PA concentrations in a body weight‐independent manner. These reductions in indole metabolites were associated with a decrease in AhR signaling and an increase in a marker of gut inflammation in RYGB‐operated mice. Overall, these data identify a potentially novel pathway by which bariatric surgery impacts gastrointestinal inflammation. A limitation of this study is that only male mice were studied. Further work is needed to understand the causative role of AhR signaling in changes in gut inflammation after bariatric surgery in humans. Further, given the impact of bariatric surgery to alter the quantity and composition of food consumption, future studies are needed to determine the impact of alterations in feeding behavior on indole profile. Correcting the AhR signaling deficiency through supplementation with indole‐producing probiotics may provide new avenues to reduce gut inflammation following RYGB.

## FUNDING INFORMATION

This work was supported by the NIDDK R00DK117509 awarded to AE, the NIEHS‐funded UC Davis EHSC under P30 ES023513 awarded to AE, The President's Council of Cornell Women awarded to BPC and NICCH R21AT010956 awarded to BPC. The content is solely the responsibility of the authors and does not necessarily represent the official views of the UC Davis nor the National Institutes of Health.

## CONFLICT OF INTEREST STATEMENT

The authors declare no conflicts of interest.

## ETHICS STATEMENT

All applicable institutional and/or national guidelines for the care and use of animals were followed. Informed Consent does not apply.

## Supporting information


Tables S1–S2.

## Data Availability

All data are available in the manuscript and the supporting materials and raw data available upon request. Further information should be directed to the corresponding authors, Dr. Bethany Cummings (bpcummings@health.ucdavis.edu) and Dr. Allison Ehrlich (akehrlich@ucdavis.edu).
