# Supplementary material for: Roux‐en‐Y gastric bypass surgery decreases intestinal aryl hydrocarbon receptor signaling in male mice
Source: Physiol Rep. 2025 Aug 26;13(16):e70524. doi: 10.14814/phy2.70524 (PMC12378703; doi:10.14814/phy2.70524)
Supplement: Supplementary file 1 — Tables S1–S2. [file PHY2-13-e70524-s001.docx]

**Supplemental Tables**

Table S1 - Differential abundance of plasma metabolites by surgical group

***RYGB vs. S-AL RYGB vs. S-WM***

*Metabolite Abbreviation Mean Diff. Adj. P Value Mean Diff. Adj. P Value*

Indole-3-acetic acid I3AA -0.0490 0.2621 -0.0337 0.5305

Kynurenic acid KA 0.0501 0.3892 -0.2090 0.0093

Indole-3-carboxaldehyde I3CAld 0.0070 0.9846 -0.0200 0.8272

Indole-3-propionic acid I3PA -0.2183 0.7930 -1.3000 0.0220

Indole-3-lactic acid I3LA 0.4121 0.2659 0.6800 0.0374

5-Hydroxyindole-3-acetic acid 5HI3AA 0.0986 0.4328 0.1654 0.1044

Kynurenine Kyn -0.0851 0.6782 -0.0866 0.3647

3-Indoleacrylic acid 3IAcrA -0.0017 0.9971 -0.0350 0.4861

2-Oxindole 2OI 0.0587 0.2017 0.0097 0.9602

Table S2 - Differential abundance of gut luminal metabolites by surgical group

***RYGB vs. S-AL RYGB vs. S-WM***

*Metabolite Abbreviation Mean Diff. Adj. P Value Mean Diff. Adj. P Value*

Indole-3-acetic acid I3AA -0.3707 0.6958 -0.7899 0.1885

Kynurenic acid KA -1.3290 0.2288 -0.0462 0.8089

Indole-3-carboxaldehyde I3CAld 1.8860 0.1338 2.4590 0.0565

Indole-3-propionic acid I3PA -0.3068 0.5174 -0.7829 0.0864

Indole-3-lactic acid I3LA -0.2548 0.6131 0.0822 0.1922

5-Hydroxyindole-3-acetic acid 5HI3AA -1.4330 0.5620 -0.2533 0.9364

Kynurenine Kyn 0.0788 0.1782 0.1109 0.0430

3-Indoleacrylic acid 3IAcrA -0.0478 0.9143 -0.3715 0.0508

Tryptamine Trptm -0.1699 0.1453 0.0119 0.8101

2-Oxindole 2OI 6.7460 0.9401 20.0600 0.5182

2,8-Dihydroxyquinoline DHQ 0.1596 0.9637 -1.6740 0.1465

Indole-3-carboxylic acid I3CA -0.3322 0.1230 -0.1294 0.6977
